# Design, implementation and evaluation of a practical pseudoknot folding algorithm based on thermodynamics

**DOI:** 10.1186/1471-2105-5-104

**Published:** 2004-08-04

**Authors:** Jens Reeder, Robert Giegerich

**Affiliations:** 1 Faculty of Technology, Bielefeld University, 33615 Bielefeld, Germany

## Abstract

**Background:**

The general problem of RNA secondary structure prediction under the widely used thermodynamic model is known to be NP-complete when the structures considered include arbitrary pseudoknots. For restricted classes of pseudoknots, several polynomial time algorithms have been designed, where the *O*(*n*^6^)time and *O*(*n*^4^) space algorithm by Rivas and Eddy is currently the best available program.

**Results:**

We introduce the class of canonical simple recursive pseudoknots and present an algorithm that requires *O*(*n*^4^) time and *O*(*n*^2^) space to predict the energetically optimal structure of an RNA sequence, possible containing such pseudoknots. Evaluation against a large collection of known pseudoknotted structures shows the adequacy of the canonization approach and our algorithm.

**Conclusions:**

RNA pseudoknots of medium size can now be predicted reliably as well as efficiently by the new algorithm.

## Background

### Biological relevance

Pseudoknots have been shown to be functionally relevant in many RNA mediated processes. Examples are the self-splicing group I introns [1], ribosomal RNAs, or RNaseP. Recently, pseudoknots were located in prion proteins of humans, and confirmed for many other species [2]. With the current increased interest in the universe of RNA functions [3], algorithmic support for analysing structures that include pseudoknots is much in demand.

### Previous algorithmic work

Well established algorithms for the prediction of RNA secondary structures (MFOLD [4], RNAfold [5]) are commonly based on a thermodynamic model [6], returning a structure of minimal free energy, called MFE-structure for short. In spite of their importance, pseudoknots are excluded from consideration by these programs for reasons of computational complexity: While folding a sequence of length *n *into unknotted structures requires *O*(*n*^3^) time and *O*(*n*^2^) space, finding the best structure including arbitrary pseudoknots has been proved to be NP-complete [7,8]. In fact, the proof given in [8] uses a scoring scheme based on adjacent base pairs only, simpler than the MFE model because it neglects entropic energies from loops. These complexity results leave two routes to achieve practical algorithms.

The first route is to consider pseudoknots in full generality, but resort to an even more simplistic energy model. An *O*(*n*^4^) time and *O*(*n*^3^) space algorithm for base pair maximization has been given in [7], and an *O*(*n*^3^) time algorithm based on maximum weight matching in [9] and [10].

The second route is the one followed here: We retain the established thermodynamic model, but restrict to a more tractable subclass of pseudoknots. For some quite general classes of pseudoknots, polynomial time algorithms have been designed: Rivas and Eddy achieve *O*(*n*^6^) time and *O*(*n*^4^) space [11]. This algorithm is available, and, in spite of the high computational cost, it is actually used in practice. We build upon this work and shall call it *pknotsRE *for later reference. Further improvements have been shown to be possible for yet more restricted classes, e.g. the non-recursive simple pseudoknots considered by Lyngsø and Pedersen [12] with *O*(*n*^5^) time and *O*(*n*^4^) space, but to our knowledge, no implementations are available. Recently, an *O*(*n*^4^) time and *O*(*n*^3^) space algorithm based on the technique of [7], that uses a thermodynamic model has been reported in [13]. While it handles simple pseudoknots consisting of more than two helices, it is restricted to non-recursive pseudoknots. Thus, this class of pseudoknots and the class presented here have a nonempty intersection, but neither of them contains the other.

### Our contributions

The new contributions reported here are the following:

• We present an algorithm *pknotsRG *for folding RNA secondary structures including pseudoknots under the MFE model which requires *O*(*n*^4^) time and *O*(*n*^2^) space.

• The algorithm considers the class of simple recursive pseudoknots, further restricted by three rules of canonization. Each simple recursive pseudoknot has a canonical representative that is recognized by *pknotsRG*.

• While this class is more restricted than the one of the Rivas/Eddy algorithm, practical evaluation shows that our algorithm finds the same pseudoknots, while the length range of tractable sequences is increased significantly.

• We provide an evaluation of the class of pseudoknots introduced here against known examples from the literature.

• We perform a rigorous evaluation of our algorithm on 212 sequences from PseudoBase [14] plus 7 other structures and compare our results with those obtained with *RNAfold *and, where feasible, with *pknotsRE*.

## Results

It is not easy to relate the classes of pseudoknots recognized by the different algorithms mentioned above. We refer the reader to the review by Lyngsø and Pedersen [8], which compares these classes by means of examples. The starting point of our work is the algorithm *pknotsRE *by Rivas and Eddy. It recognizes pseudoknots that can be nested and can have unlimited chains of helices involved in crosswise interactions. The drawback of this powerful, but computationally expensive algorithm is the following paradox: Pseudoknots with complex helix interactions naturally require longer primary sequence than simpler ones. The high runtime complexity of *O*(*n*^6^), however, as well as the space consumption of *O*(*n*^4^) restricts the use of this algorithm to a maximal sequence length of around 150 nucleotides. Most of the pseudoknots predicted belong to a much simpler structural class and do not exhibit chains of crosswise interactions.

The algorithm developed here achieves time complexity *O*(*n*^4^) and space complexity *O*(*n*^2^). The runtime improvement, compared to *pknotsRE*, results from an idea of canonization, while the space improvement results from disallowing chained pseudoknots. These improvements extend the range of tractable sequences to a length up to 800 nucleotides, and we can locate pseudoknots up to this size in even longer sequences.

### Simple recursive pseudoknots

**Figure 1:**
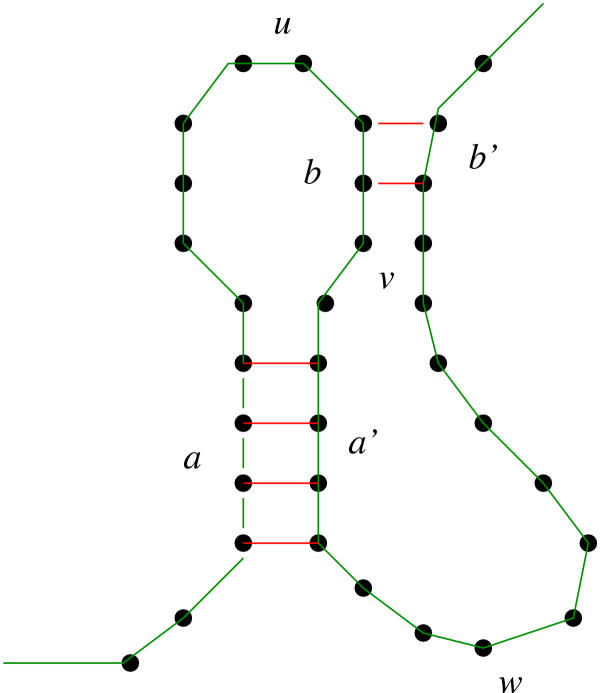
**A simple pseudoknot. **A simple pseudoknot, formed by helices *a – a' *and *b – b'*, with intervening sequences *u, v, w*. If the internal parts *u, v, w *contain further secondary structures, we call it a simple recursive pseudoknot.

Following the terminology of [7], a *simple *pseudoknot is a crosswise interaction of two helices, as shown in Figure 1. In simple *recursive *pseudoknots, we allow the unpaired strands *u, v, w *in a simple pseudoknot to fold internally in an arbitrary way, including simple recursive pseudoknots. Let us call this class sr-PK. More complex knotted structures like triple crossing helices or kissing hairpins, as shown in Figure 4, are excluded from sr-PK. We will show later how they can be integrated in our approach and outline the increased computational cost of doing so. For the main part of this paper, we concentrate on the class sr-PK.

### Anticipating the complexity of a DP algorithm

Thermodynamic RNA folding is implemented via dynamic programming (DP). We start with a semi-formal discussion of how to estimate the efficiency of a DP algorithm for folding (or any kind of motif search) *before *it is written in detail. We consider elements of RNA structure as sequence motifs of different types: hairpins, bulges, multiloops, etc. The following notation is taken from the algebraic dynamic programming approach [15]. By an equation

m = f <<< a ~~~ b ~~~ c | | | g <<< c ~~~ a

we specify that the sequence motif *m *can be composed in two alternative ways: The first case, labelled by *f*, requires adjacent occurrences of motifs *a*, *b *and *c*. The second case, labelled by *g*, requires adjacent occurrences of motifs *c *and *a*. When motif *m *is to be scored, *f *and *g *are seen as the scoring functions that combine the local score contribution of each case with the scores of sub-motifs *a*, *b*, and *c*.

What is the computational effort of locating motif *m *in an input sequence *x *of length *n*, say at sequence positions *i *through *j? *First we assume that all motifs can have arbitrary size between 0 and *n*. The algorithm must consider all boundary positions (*i, j*) for motif *m*, which requires *O*(*n*^2^) steps at least. In case *g*, it must consider all boundary positions *k *where motifs *c *meets *a*, such that the runtime for case *g *is in *O*(*n*^3^). In case *f*, there are two such moving boundaries *k *and *l *between the three sub-motifs, so we obtain *O*(*n*^4^) overall for motif *m*.

This can be improved if there is an upper bound on the size of some motif involved. If motif *a *is a single base, for example, the exponent of *n *decreases by 1 in both cases. Furthermore, if motif *b *is (say) a loop of maximal size 40, then one factor of *n *is reduced to a constant factor and overall asymptotic runtime is now *O*(*n*^2^). Sometimes a motif description can be restructured to improve efficiency by reducing the number of moving boundaries. Whether or not this is possible does not depend on the motif structure, but on the scoring scheme! This is a somewhat surprising observation from [15], where such optimizations are studied, and where also the line of reasoning exercised here is given a mathematical basis.

In the sequel, we shall exploit another source of efficiency improvement. If the lengths of two sub-motifs are coupled somehow, say *a *and *c *have the same length, then the boundaries *k *and *l *in case *f *are related by *k *- *i *= *j *- *l*. When iterating over *k*, we can use *l *:= *j *- *k *+ *i *(rather than *k *≤ *l *≤ *j*)and save another factor of *n*.

### Canonization

When the search space of a combinatorial problem seems to be too complex to be evaluated efficiently, heuristics are employed. Canonization restricts the search space in a well-defined way, arguing that all the relevant solutions in the full search space have a representative that is canonical, and hence, nothing relevant is overlooked. One such technique is the purging of structures that have isolated basepairs. Here the plausibility argument refers to the underlying energy model, where base pairings without stacking have little or no stabilizing effect. This canonization does not affect efficiency, but it achieves a significant reduction of the search space (figures in [16]), which renders the enumeration of near-optimal solutions [17] much more meaningful.

**Figure 2:**
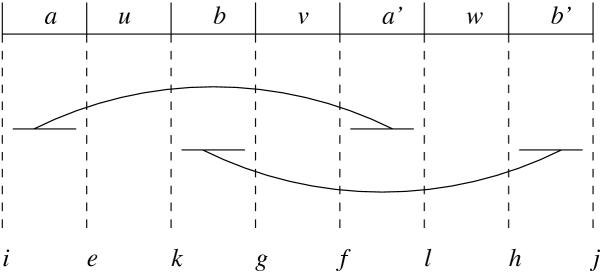
**The boundaries of a pseudoknot. **Eight moving boundaries delineating a simple recursive pseudoknot.

We shall introduce three canonization rules that reduce class sr-PK to the class of *canonized simple recursive pseudoknots*, csr-PK. Using the notation introduced above, the motif definition of a simple recursive pseudoknot is given by

knot = knt <<< a ~~~ u ~~~ b ~~~ v ~~~ a' ~~~ w ~~~ b'

with boundaries at sequence positions *i, e, k, g, f, l, h, j *as shown in Figure 2.

Segment *a *forms a helix with *a*', and *b *with *b'*. Segments *u*, *v*, and *w *can have arbitrary structures, including pseudoknots. Naively implemented, we can expect a DP algorithm of time complexity *O*(*n*^8^) according to our efficiency estimation technique introduced above. We now apply canonization. Note that it only applies to helices forming pseudoknots; other helices are unaffected. We first present the technical aspects; the discussion of these restrictions is deferred to the next section.

#### Canonization rule 1

(a) Both strands in a helix must have the same length, i.e. |*a*| = |*a*'| and |*b*| = |*b*'|. (b) Both helices must not have bulges.

Note that (b) is a stronger restriction and trivially implies (a). Under the regime of Rule 1 we may conclude:

*f *= *l *- (*e *- *i*)

*h *= *j *- (*g *- *k*)

We are left with 6 out of 8 boundaries that vary independently, and runtime is down to *O*(*n*^6^).

#### Canonization rule 2

The helices *a*, *a' *and *b*, *b' *facing each other must have maximal extent, or in other words, compartment *v *must be as short as possible under the rules of base pairing.

We observe that the maximal length of *a *and *a' *is fixed once *i *and *l *are chosen. The maximal helix length *stacklen*(*i, l*) can be precomputed and stored in an *O*(*n*^2^) table. The same observation holds with respect to the other helix, and we fix

*e *= *i *+ *stacklen*(*i, l*)

*g *= *k *+ *stacklen*(*k, j*).

Thus, we are left with only four independently moving boundaries – *i, k, l, j *–, and can hope to obtain an algorithm with runtime O(*n*^4^). Scores of pseudoknots found between *i *and *j *are stored in table *knot*(*i, j*), and hence the space requirements are O(*n*^2^), which is the same asymptotic space efficiency as in the folding of unknotted structures.

A subtlety arises when both helices, chosen maximally, compete for the same bases of *v*, or in other words, the length of *v *would become negative. This case is addressed by

#### Canonization rule 3

If two maximal helices would overlap, their boundary is fixed at an arbitrary point between them.

Let *m *and *m' *be the helix lengths so determined. We finally obtain

*e *= *i *+ *m*

*g *= *k *+ *m'*

The language of pseudoknots in class csr-PK can be defined by a simple context free grammar over an infinite terminal alphabet. Let *a*^*k *^denote a terminal symbol of *k *times the letter *a*. The grammar uses a single nonterminal symbol *S *and its productions are


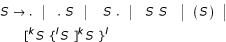


for arbitrary *k, l *≥ 1. For example, the simple pseudoknot of Figure 1 is represented as the string

.. [[[......{{..]]]]..........}}.

This grammar is useful to judge how different an experimentally determined structure is from class csr-PK. It is not useful for programming, since it is ambiguous and does not distinguish the fine grained level of detail required in the energy model.

### Canonical representatives

A careful discussion is required to show that each simple recursive pseudoknot, if not canonical by itself, has (a) a canonical representative of (b) similar free energy.

Rule 1 (b) affects the length of helices that are considered in forming the pseudoknot. Let there be a pseudoknot between *i' *and *j'*. It is not canonical if one of the two helices contains bulges. However, there must be at least one pair of shorter helices without bulges at *i, j *with *i' ≤ i *and *j *≤ *j'*, which serves as a canonical representative, albeit with somewhat higher free energy.

Rule 2 is justified by the fact that the energy model strongly favours helix extension. Clearly, for each family of pseudoknots delineated by *i, k*, *l*, *j *there is a canonical one with maximal helices, whose free energy is at least as low – except for the following case: The maximal helices compete with the internal structure of *u*, *v *and *w*. It may be possible to contrive a structure where shortening (say) helix *a – a' *by one base pair allows to create two pairs with new partner bases in *u *and *v*, resulting in a structure which has slightly lower energy. Still, the free energy of the canonical pseudoknot must be very similar.

Finally, Rule 3 requires a decision where to draw the border between two helices facing each other and competing for the same bases. An arbitrary decision here can only slightly affect free energy, as the same base pairs are stacked either on the a – *a' *or the *b – b' *helix.

Let *E*(*s*) denote the free energy computed for structure *s*. Summing up, we have shown that for each simple recursive pseudoknot *K*, there is a canonical one *C *in the search space. While we cannot prove that *E*(*C*) ≤ *E*(*K*), we have argued that this is likely, and if not, the energies will at least be close. Still, there might be another, energetically optimal canonical structure *S *(knotted or not) such that *E*(*K*) <*E*(*S*) <*E*(*C*). In this case, if only the "best" structure *S *is reported, neither *K *nor its canonical representative *C *is observed. (A remedy to this is the computation of near-optimal structures.)

Finally, let us add that the implementation described below is actually slightly more general that the "pure" csr-PK model described above: We do allow a single nucleotide bulge in either helix of a pseudoknot, which complicates the program, but does not affect asymptotic efficiency.

### Evaluation of the class csr-PK

To evaluate how well the class csr-PK covers known pseudoknots, we considered 212 pseudoknot structures from PseudoBase. The observations are shown in Table 1.

We find 172 simple recursive pseudoknots, and 40 of more general shapes. We find that 135 out of the 172 pseudoknots lie in csr-PK, i.e. they are their own canonical representatives. 11 more fall into the relaxed csr-PK, where we allow a single nucleotide bulge in Canonization Rule 1. Thus, we cover 146 out of 212 (68%). 26 simple recursive pseudoknot do not fall in class csr-PK, since they contain isolated basepairs, non canonical basepairs or one of the helices has not maximal extent.

**Figure 3:**
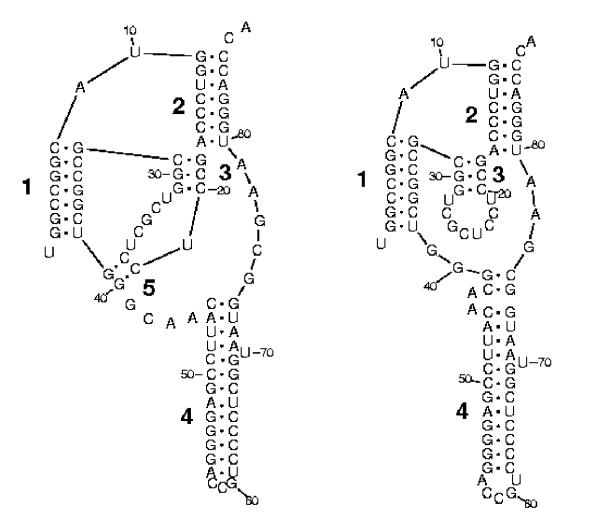
**The pseudoknot of Hepatitis Delta Virus. **On the left side the structure proposed by [32], on the right the MFE-structure predicted by *pknotsRG*. Our algorithm misses only the short helix 5 and adds 3 basepairs to helix 4. Image created with RnaViz2 [33].

Considering the remaining 20% complex pseudoknots, note that often pseudoknots in more general classes also have a good representative in csr-PK. For example, the pseudoknot of Hepatitis delta virus (Figure 3) consists of four interacting helices of shape *a – b – c – d – c' – a' – d' – b*', where helix *d – d' *is very short – only two base pairs. Deleting it, helix *c – c' *is no longer interacting with other helices, and the pseudoknot falls within class csr-PK.

### Better than optimal 

There are many reasons why "the" MFE structure may only be part of what we want to know about a molecule's foldings. To deal with the problem when the optimal (knotted) structure is non-canonical, and its canonical representative is dominated by an unrelated structure, we provide two means: First of all, our algorithm is non-ambiguous, the prerequisite for a non-redundant enumeration of near-optimal structures [16]. We can let the program to report the *k *best structures. Secondly, we shall provide three variants of our program:

*pknotsRG-mfe *computes the mfe structure (or the k best), pseudoknotted or not.

*pknotsRG-enf *picks out from the folding space the energetically best structure that contains at least one pseudoknot.

*pknotsRG-loc *computes the energetically best pseudoknot that can be formed locally, i. e. somewhere in the sequence. "Best" is defined here as minimal free energy per base, to avoid a built-in bias towards large pseudoknots.

The best local pseudoknot motif is included by adding two cases:

bestPK = skipleft <<< base ~~~ bestPK ||| bestPKl

bestPKl = skipright<<< bestPKl ~~~ base ||| knot

These clauses have time complexity *O*(*n*^2^) and preserve the non-ambiguity of the algorithm. If desired, an enumeration of near-optimal "local" pseudoknots is also feasible.

### Predictive accuracy

We first consider the predictive accuracy achieved by our approach. We have already evaluated the class csr-PK against the known pseudoknots, and we know that our algorithm correctly implements this class in its search space. What is really tested in the following is the adequacy of the current thermodynamic model (which our algorithm shares with *RNAfold *and in an older version with *pknotsRE*), and the results in this section may improve if this model is further improved in the future.

We test our algorithm on the set of sequences listed in Table 2, including 212 sequences from PseudoBase. Although there is some redundancy on the sequence level, there is a good reason why we found it important to use all available sequences for testing: Even near identical sequences can have different MFE structures, or a small change may prevent successful pseudoknot prediction. In contrast to [13] we did not restrict the evaluation to the class of pseudoknots recognized by our program. It is also instructive to retain the difficult cases, and see whether the predictions catch at least some aspect of a more general pseudoknot.

We compare our results to the output of *RNAfold*, as a representative for RNA folding tools without pseudoknot folding capability, and to *pknotsRE *where computationally feasible. For each predicted structure we count the number of correctly and falsely predicted base pairs (TP and FP). Let BP be the number of basepairs in the reference structure from the database or literature. We define the sensitivity as (TP/BP), selectivity as (TP/TP+FP).

In Table 3 we list the prediction accuracy for our sequence set. For all sequences we enhance the prediction accuracy with respect to *RNAfold*. Both, the sensitivity and the selectivity increase. Compared to *pknotsRE *our results are slightly better, probably because we are using the newer and subtler energy model. For example, for the sequence of hepatitis delta virus, our algorithm predicts all helices except for the very short helix 5 (see Figure 3), while the other programs miss more than 50% of the basepairs.

We also folded 14 randomly selected human tRNAs (third line in Table 3) and found only one false positive pseudoknot. Interestingly, the pseudoknotted structure has two helices (9 bp) in common with the true clover-leaf structure, while the structure computed by *RNAfold *has only one helix (4 bp). For all programs the overall prediction accuracy for tRNAs is not very high. tRNAs are a known hard case for structure prediction because they contain many modified bases.

Since we use the same energy model as *RNAfold *and our algorithm does not introduce spurious pseudoknots, predictions of *RNAfold *and *pknotsRG *for unknotted structures are identical. Of course, if there is more than one optimal structure, each of the optimal alternatives may be reported and thus the same folding can not be guaranteed.

### Computational performance

Clearly, we are able to fold sequences that are longer than *pknotsRE's *limit of 150 nucleotides. Short sequences up to 100 nucleotides are folded within a minute. Long sequences (400 bp) take about 2 hours (see Table 4). If we restrict the maximal pseudoknot size to a reasonable constant, say 150 nucleotides, we can further increase the running time. The algorithm runs now in *O*(*cn*^3^) with a rather large constant *c*. This enables us to fold sequence of length 1000 in approximately 12 hours. We can further observe that the space requirements scale quadratically with the input size, as expected.

For a fair comparison, the reader should keep in mind that the extra time spent by *pknotsRE *is not strictly wasted: It is spent on assuring that the optimal folding of the input RNA sequence does not contain pseudoknots with chained interacting helices of lower free energy than the reported structure. *pknotsRG *does not consider such structures and hence cannot make this assertion.

## Discussion

In the following, we discuss extensions of the implemented model and their expected computational cost

### Bulges, triple crossing and kissing hairpins

Canonization Rule 1 can be relaxed further to allow larger bulges inside the helices forming a pseudoknot. As long as their number (and hence the length difference of the two arms of a helix) is bounded by a constant, asymptotic efficiency is not affected.

**Figure 4:**
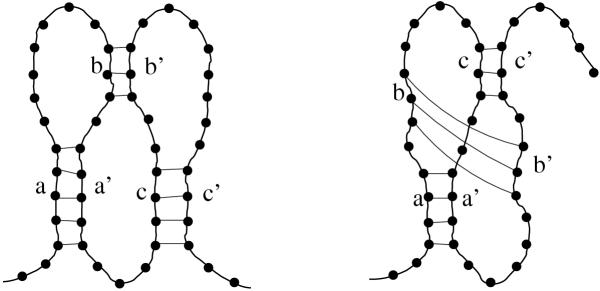
**Kissing hairpins and triple helix interaction. **Two examples of more complex pseudoknots with three helices: Kissing hairpins (left) and triple helix interaction (right).

Two examples of non-simple pseudoknots are shown in Figure 4. We can incorporate them into our algorithm adding the definitions

kiss = kss <<< a~~~u~~~b~~~v~~~a'~~~w~~~c~~~x~~~b'~~~y~~~c'

triple = trp <<< a~~~u~~~b~~~v~~~c~~~w~~~a'~~~x~~~b'~~~y~~~c'

Canonization can be applied as above, with Rule 3 becoming more sophisticated for the triple interaction case. This would yield an algorithm of runtime *O*(*n*^6^), bringing runtime back to the efficiency class of the Rivas/Eddy algorithm. But note that the space requirements remain *O*(*n*^2^). This is due to the fact that we now consider three interacting helices, but not arbitrary chains.

### Folding long sequences

RNA folding *in vivo *as *in vitro *must be understood as a hierarchical process, where small structures in close vicinity form first, and then combine to larger ones [18]. The folding path becomes relevant, and the longer a sequence, the more unlikely it is that its folding path leads to a global energy minimum. In other words, the longer the sequence, the less reliable are the results of minimum free energy folding. *pknotsRG *gives us the possibility to test this using a fairly large structure containing pseudoknots that have been proved experimentally. We considered the sequence of the group I intron from Tetrahymena thermophila (419 NT) (V01416).

The MFE-structure found was quite different from the "true" structure taken from the literature. We hand-coded the experimental structure and evaluated its stability in our energy model. The result was striking: the experimental structure (-132.26 kcal/mole) was significantly far from the possible minimum of free energy (-155.64 kcal/mole). So far in fact that it seems infeasible to detect the structure by scanning the space of near-optimal structures. This could be interpreted as the energy model being incorrect, but since it works well for short sequences, we suggest that this is an indication that the kinetics of folding already have a strong influence with this size of sequence, at least when pseudoknots are involved.

While we have achieved a considerable speedup for predicting small pseudoknotted structures, it seems that minimum free energy approach is not meaningful with the largest structures which it now can handle algorithmically. However, the situation changes when we are looking for particular structural motifs (see below).

## Conclusion

We presented an algorithm *pknotsRG-mfe*, based on the MFE-model, for finding the best RNA structure including the pseudoknot class csr-PK. This requires *O*(*n*^4^) time and *O*(*n*^2^) space. The algorithm variant *pknotsRG-enf *returns the energetically best structure that contains a pseudoknot (interesting when the MFE structure is unknotted), while *pknotsRG-loc *reports the best pseudoknot (under a length-normalized energy score) *somewhere *in a sequence. We achieve a high prediction accuracy for moderate length sequences, whereas long sequences, at least when pseudoknots are involved, seem to have a folding scheme that cannot be modelled with minimum free energy folding.

Algorithm *pknotsRG *is based on a simpler grammar model than the crossed interaction grammars [19] underlying *pknotsRE*, as well as the communicating grammars underlying the recent approach by Cai [20]. It requires only a minor extension over the ADP tree grammars that are applicable to a wide range of sequence analysis problems [21]. Furthermore, the grammar is not only a theoretical backup, explaining the underlying model. With minor annotation for the sake of efficiency, the grammar actually constitutes executable code. This means that *pknotsRG *can serve as a template for a new class of programs we call thermodynamic matchers.

Many functionally important RNAs like RNase P or group-I-introns have known structures that include pseudoknots. The search for such motifs using combinatorial matchers like RNAmotif [22] is hampered by the problem that a motif description is either too specific and misses relevant instances, or else it is too vague and produces a large number of different matches to the same sequence. We suggest to develop *thermodynamic matchers*, which are RNA folding programs, based on the established MFE model, but specialized to the particular structural motif at hand. Such a matcher returns the optimal way to fold a sequence into the motif structure, together with the free energy of this folding. Comparing this energy to the MFE of an unrestricted folding can give us a hint with respect to the significance of such a match.

## Methods

### Choice of implementation method

Using the ideas presented so far, our folding algorithm can be implemented in any language suitable for dynamic programming, say FORTRAN or C. However, we are interested in a reusable implementation that can be integrated without change in specialized folding programs called thermodynamic matchers. Therefore *pknotsRG *was implemented using the method of algebraic dynamic programming (ADP) [15,23].

### RNA folding in ADP

In ADP, the search space of a DP problem is defined on a declarative level, specified by clauses like the ones we have already seen above. Together they form a tree grammar, defining a tree language whose elements are all the candidates in the search space. In our case, the candidates are RNA structures represented as trees. The typical DP recurrences are implicit in this description. Scoring is achieved by interpreting the operators (e.g., *knt, skipleft, skipright*) that build the trees as scoring functions. The grammar needs to be annotated with respect to tabulation and the application of the objective function (in our case, minimization).

The advantage of this method is its high level of abstraction. No subscripts, no errors. The perfect separation of search space definition and evaluation allows the same grammar to be used for different kinds of analyses, e. g. folding space statistics. Relevant algorithmic properties such as non-ambiguity and efficiency can be studied on this level of abstraction. Last not least, an ADP program can be executed as is, avoiding the explicit formulation of DP recurrences (and a whole universe of programming errors). A significant, but constant factor of speedup can be gained by explicitly formulating the recurrences and implementing them in a lower level language. Automating this process is part of our current work.

We start from an ADP algorithm for folding RNA secondary structures (excluding pseudoknots) provided by Dirk Evers [24]. We show ADP clauses defining the closed substructures: stacks, hairpins, bulges, and multiloops, adding an alternative for pseudoknots. region denotes an arbitrary sequence of (unpaired) bases.


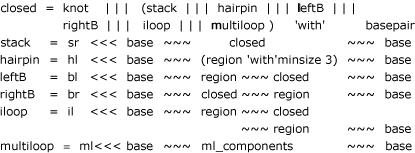


The shown code abstracts from efficiency annotation and the treatment of dangling bases. The complete algorithm is found on the ADP WWW pages [25]. It is based on the standard MFE model with dangling bases, is non-ambiguous and requires *O*(*n*^3^) time and *O*(*n*^2^) space. A size constraint of 30 is used to bound loop length in internal loops. Closed substructures are defined such as to avoid lonely base pairs. While all this is easily expressed within the standard ADP framework, our new algorithm requires extensions which are now explained.

### Adding pseudoknots

The implementation strictly follows the outline given in the methods section, except that a considerable amount of detail related to the energy model has to be taken care of. While ADP bans the use of subscripts, our canonization ideas require to explicitly manipulate subscripts. We show the concrete pseudoknot code, but explain only the essential points. A subscript pair (*i, j*) denotes input sequence positions *inp*_*i*+1_.. .*inp*_*j*_. [...] denotes lists, and <- denotes enumerating a list of alternative values.

knot (i, j) = [pk energy a u b v a' w b' | k <-[i+2 .. j-1], l<-[k+1 .. j-2],

These line chooses *k *and *l *from the interval (*i, j*), and put together the results from *a*, *u*, *b*, *v, a'*, *w, b' *under the scoring function *pk*. Each helix must have a minimum length of two bases. Due to stereochemical reasons one base in the front part and two bases in the back part are left explicitly unpaired; these bases should bridge the stacks. This consideration is taken over from *pknotsRE*. The next definitions implement canonization rules 1, 2 and 3. They determine the helix lengths, finally computed into the variables *m *and *m'*. If either of them is smaller than 2, a pseudoknot is not possible at this particular location.


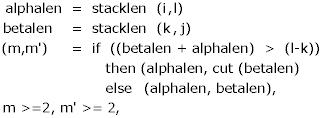


The function *cut *shortens the helix *b – b' *as much as necessary in case of overlapping helices. The next lines define the pseudoknot components *a *through *b*', plus the local energy contribution. To avoid an extra factor of *n *in time complexity, the energies of maximal length helices are also precomputed in table *stackenergy*. If the helix *b – b' *must be chosen shorter than maximal to avoid overlap, a correction term has to be subtracted. This explains the negative term in the energy computation.


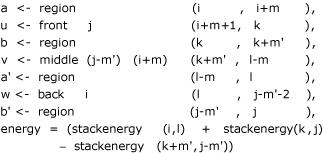


Left to be defined are the interior structures front, middle, and back. For reasons of space, we only show the definition of *front*. For a full implementation of the algorithm see additional file 1.


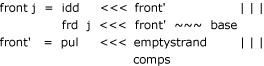


This case takes care of a potentially dangling base from the *b*-helix, and if the remaining region is not empty, an arbitrary list of substructures (*comps*) is recognized. *idd, frd *and *pul *are the corresponding functions from the energy model.

Overall, the energy of a pseudoknot consists of stabilizing and destabilizing terms. Where possible, we use the values from the current thermodynamic energy model [6]. As stabilizing terms we count the nearest neighbour stacking energies of the pseudoknot helices and contributions of dangling bases at both ends of each helix. If the length of the middle part *v *is smaller or equal to 1, the pseudoknot helices stack coaxially on each other and we further add the appropriate stacking energy. In [11] a pseudoknot initiation parameter of 7 kcal/mole is proposed. However, we found out, that setting this value to 9 kcal/mole performs better with the new energy model. Our observation supports the similar choice made by Dirks and Pierce [26]. Finally, we penalize each unpaired nucleotide inside a pseudoknot loop with 0.3 kcal/mole. This seems to be the best approximation of the values given in [27]. Of course, if the pseudoknot is recursive the energy of the subcomponent is taken into account as well.

The first clause (knot) chooses *k, l *inside (*i, j*), computes *m *and *m' *using the precomputed maximal helix information, and passes these boundaries to the pseudoknot compartments. Methodically, this is a use of inherited attributes with the underlying tree grammar, and appears to be a novel technique in dynamic programming, at least in its grammar oriented tradition [19,28-30].

The relative effort of implementing the three variants of *pknotsRG *can be judged from the sizes of the tree grammars required, which are summarized in Table 5.

### Availability

The three variants of the algorithm *pknotsRG-mfe, pknotsRG-enf*, and *pknotsRG-loc *are available as executables and source code on the Bielefeld Bioinformatics Server [31].

## Authors' contribution

RG had the initial idea for the algorithm. JR developed and evaluated the software. All authors read and approved the final manuscript.

## Supplementary Material

Additional File 1 Source code of pknotsRG-mfe Click here for file

## Figures and Tables

**Table 1:** Class membership of 212 pseudoknots from PseudoBase. The sequences were determined by comparative sequence analysis and/or by experimental techniques. The largest class of pseudoknots is simple recursive or even canonical simple recursive.

simple recursive pseudoknots					
csr-PK	1-nt bulge	Rule 2 violated	isolated basepair	G-A basepair	total

135	11	6	17	3	172

complex pseudoknots					

internal loop	triple helix	four helices	kissing hairpins	large bulge	total

23	12	1	3	1	40

**Table 2:** Sequences used for testing

Sequence	Length	BP	Reference
PseudoBase	variable	variable	[14]
7 HIVRT	35	11	[34]
14 tRNAs	71–82	18–19	
HDV	87	32	[32]
ag-HDV	91	25	[32]
TYMV	86	24	[35]
TMV (up)	85	25	[36]
STNV	252	69	GenBank:M64479

**Table 3:** Evaluation of predictive accuracy *TP *= true positives, sens. = sensitivity, *FP *= false positives, sel. = selectivity, *K *= (number of correct predicted pseudoknot helices)/ (expected number of pseudoknot helices). False negatives can be derived from: *FN *= *BP *– *TP*. For HIVRT, PseudoBase and tRNA the average over all sequences is taken.

		RNAfold			pknotsRE			pknotsRG-mfe		
Sequence	BP	*TP*(sens.)	FP(sel.)	K	TP(sens.)	FP(sel.)	K	TP(sens.)	FP(sel.)	K
PseudoBase	13.1	7.1(54.2)	4.4(61.9)	-	9.2(72.3)	3.8(70.7)	-	10.2(77.3)	3.5(74.7)	-
HIVRT	11	4.7(42.8)	1.7(73.3)	1/2	11(100)	0(100)	2/2	11(100)	0(100)	2/2
tRNAs	18.7	9.4(50.4)	12.5(43.0)	0/0	-	-	-	9.8(52.3)	12.2(44.5)	0/0
HDV	32	12(37.5)	16(42.9)	2/4	14(43.8)	16(46.7)	2/4	29(90.6)	2(93.5)	3/4
ag-HDV	25	4(16.0)	24(14.3)	1/2	24(96.0)	9(72.7)	2/2	21(84.0)	11(65.6)	2/2
TYMV	24	17(70.8)	6(73.9)	1/2	24(100)	1(96.0)	2/2	23(95.8)	2(92.0)	2/2
TMV	25	13(52.0)	8(61.9)	3/6	13(52.0)	9(59.1)	3/6	20(80.0)	4(83.3)	6/6
STNV	69	26(37.7)	54(32.5)	2/8	-	-	-	42(60.9)	37(53.2)	5/8

**Table 4:** Performance results Performance results for random RNA sequences and comparison to *pknotsRE*. The performance of *pknotsRG-loc *is similar to *pknotsRG-mfe *and therefore not shown. The bounded version restricts pseudoknots to a maximum length of 150 nucleotides; measured on an UltraSPARC CPU with 450 MHz and 4 GB main memory. Time is measured in (h:m:s), memory in MB.

Length	pknotsRE		pknotsRG-mfe		pknotsRG-enf		pknotsRG-enf bounded	
	Time	Mem.	Time	Mem.	Time	Mem.	Time	Mem.
40	17.4	-	0.7	1	0.9	2	0.9	2
80	21:11	38	9.5	5	8.7	5	8.8	5
100	1:23:50	80	20.0	8	32.5	10	34.5	9
200	-	-	6:46	36	8:03	42	6:33.6	42
300	-	-	33:07	80	44:58	102	20:48.4	90
400	-	-	1:48:08	146	2:28:18	184	45:28.9	155

**Table 5:** Program sizes

Program variant	Nonterminals	Productions	Tables
pknotsRG-mfe	27	74	8
pknotsRG-loc	27	68	8
pknotsRG-enf	40	119	11
